# The Role of Sense of Power in Alleviating Emotional Exhaustion in Frontline Managers: A Dual Mediation Model

**DOI:** 10.3390/ijerph17072207

**Published:** 2020-03-25

**Authors:** Song Liu, Hao Zhou

**Affiliations:** Business School, Sichuan University, 29 Wangjiang Road, Chengdu 610064, China; liusong@stu.scu.edu.cn

**Keywords:** affective commitment, emotional exhaustion, managerial self-efficacy, sense of power, survey study

## Abstract

Frontline managers have many responsibilities and often suffer from emotional exhaustion. Drawing on the job demands–resources model, this research proposes and examines a cognitive–affective dual mediation model to explain how frontline managers’ sense of power affects their emotional exhaustion through managerial self-efficacy (cognitive path) and affective commitment (affective path). A cross-sectional study design was employed, and the theoretical model was tested using a three-wave survey among 227 on-the-job Master of Business Administration (MBA) students (52.86% male) in China, who serve as frontline managers in different kinds of organization. The regression and bootstrapping analysis results showed that the frontline managers’ sense of power was significantly negatively related to emotional exhaustion. In other words, the more powerful they felt, the less exhausted they felt. Furthermore, having a sense of power enhanced managerial self-efficacy, which mitigated emotional exhaustion. Sense of power also boosted frontline managers’ affective commitment, alleviating emotional exhaustion. We conclude with a discussion of this study’s theoretical and practical contributions and future research directions.

## 1. Introduction

Frontline managers are the backbone of an organization, and must perform their duties under increasingly uncertain conditions including global organizations and rapid and continuous market changes. Simultaneously, with organizations becoming flatter, the wider spans of control aggravate the management difficulties of frontline managers. These factors create significant pressure and may threaten their physical and mental health, leading to serious job burnout [1,2,3,4]. “Burnout” is a widely studied topic in occupational health psychology, and refers to a state of physical and mental fatigue related to negative attitudes toward work. Burnout is characterized by three components: emotional exhaustion, depersonalization, and diminished personal accomplishment [5,6]. 

Many previous studies have found that emotional exhaustion, a chronic feeling of emotional and physical depletion resulting from workplace stressors, is a core dimension of burnout [7,8,9]. Many researchers have focused on the issue of emotional exhaustion, revealing its negative consequences such as damage to physical health, decreased job satisfaction, poor performance, and an increase in turnover [9,10,11]. Previous studies have found that, compared with ordinary employees, frontline managers bear onerous job demands (e.g., heavy workload and emotional demands), high role stress [12], and severe emotional exhaustion. However, few studies have focused on the emotional exhaustion of frontline managers, highlighting the need to further understand the antecedent and mechanism of emotional exhaustion in this specific group.

To prevent the destructive consequences of emotional failure, numerous studies have centered on the antecedents of emotional exhaustion. These factors may be personal (e.g., personalities; [11]) and situational (e.g., perceived organizational support; [13]). Some studies have found that empowering employees by providing access to support, resources, autonomy, and learning opportunities can enhance their ability to complete work efficiently and may prevent emotional exhaustion (e.g., [1,14,15]). From this perspective, frontline managers generally have specific sources of power (i.e., defined as control over valued resources; [16]) compared with subordinates. This may help alleviate emotional exhaustion. Previous studies have found that a manager’s sense of power, which is the perception of being able to influence others [17], may better explain and predict behaviors (e.g., voice) and emotions, compared with actual power [18,19,20]. Therefore, this study links frontline managers’ sense of power to emotional exhaustion.

The job demands–resources (JD-R) model [21] provides a useful theoretical framework to explain our primary argument that sense of power is a core resource enabling managers to cope with heavy job demands (e.g., critical tasks and role stress), thereby relieving emotional exhaustion. To explore the internal mechanism driving how sense of power impacts emotional exhaustion, we propose a dual mediation model with a cognitive–affective dual path. Specifically, previous research proposes that feeling powerful gives individuals confidence in making decisions [22], controlling over job outcomes [23], and perceiving optimistic to risks [24]. Hence, for the cognitive path, we drew on self-efficacy theory [25,26] to argue that managers with an elevated sense of power generally have high managerial self-efficacy. This is defined as the extent to which supervisors perceive themselves as competent and efficient in implementing their managerial roles [27,28]. This helps them confidently accomplish tasks and reduces their occupational stress and emotional exhaustion. Additionally, prior work suggests that employees’ emotional exhaustion may decrease as employees are more affectively committed to the organization [29,30]. 

In light of this, for the affective path, we applied social exchange theory [31] to propose that frontline managers with an enhanced sense of power tend to perceive that they receive respect, support, care, and other benefits from the organization. As a result, they are obliged to reciprocate with a commitment to the enterprise. This reflects affective commitment, which is one’s emotional attachment through feelings of loyalty and pride to an organization [32,33,34]. In turn, affective commitment enables frontline managers to further develop greater social exchange relationships within the organization. This satisfies primary needs and effectively fulfills managerial roles, decreasing the impact of occupational stress and emotional exhaustion.

This research contributes to the literature in three significant ways. First, our findings contribute to the emotional exhaustion literature by applying the JD–R model [21] with frontline managers and demonstrating the effect of sense of power on emotional exhaustion. Second, by examining the cognitive–affective dual path, we propose and test a dual mediation model to assess how sense of power negatively and significantly affects emotional exhaustion through managerial self-efficacy and affective commitment. This approach helps explain the internal mechanism involved. Third, the present study contributes to the power literature by centering on a personal sense of power and extending its consequences to factors such as emotional exhaustion, managerial self-efficacy, and affective commitment.

## 2. Theoretical Review and Hypotheses

### 2.1. Emotional Exhaustion

Burnout is a psychological response to chronic occupational stressors and has become a global epidemic in the workplace [4,35]. Emotional exhaustion, the primary manifestation of burnout, is defined as a chronic feeling of emotional and physical depletion caused by superabundant job demands [3,4]. Previous studies have found that emotional exhaustion, compared with depersonalization and diminished personal accomplishment, is a consistent sub-dimension of burnout in relationships with antecedent or outcome variables [7,36]. Many studies have found that emotional exhaustion exhibits a plethora of harmful consequences to both individuals and organizations [36]. For example, individuals with emotional exhaustion are prone to chronic diseases [37], reduced job satisfaction, lower job performance [10], diminished organizational loyalty [38], and higher absenteeism [11]. Prior work has primarily focused on the emotional exhaustion of enterprise employees, teachers, and medical staff (e.g., [9,39,40]). Few studies have involved the problem of emotional exhaustion of frontline managers. Therefore, this study concentrates on that specific problem.

Previous studies suggest that antecedents of emotional exhaustion focus on personal factors such as gender and neuroticism [41,42], and situational factors such as work overload and role conflict [10,43]. Nevertheless, prior research has not fully examined the correlation between sense of power, a crucial individual factor affecting personal behavior, emotions, and cognition [44], and emotional exhaustion. Accordingly, this study focused on the relationship between frontline managers’ sense of power and their emotional exhaustion, and explored its internal mechanism.

### 2.2. Impact of Frontline Managers’ Sense of Power on Emotional Exhaustion

Generally, power refers to a person’s control over precious resources such as money, information as well as appreciation, and has a profound influence on individuals [17,45]. Having power brings individuals increased job security and better financial incentives, motivates them to work more effectively, and leads to the experience of more positive affect [16,46,47]. Power is also classically defined as the perception of one’s ability to affect others. This definition has been developed into the concept of sense of power [17,48]. The primary antecedents of a personal sense of power are sociostructural factors (e.g., social positions and status), and personal characteristics (e.g., extraversion and conscientiousness). These play a vital role in determining how powerful an individual perceives himself or herself to be [17]. Previous studies have found that sense of power affects individuals’ behavior, emotions, and cognition more than objective power such as formal authority [18,44]. Individuals vary in their personal subjective perceptions of power [19]. For example, compared with ordinary employees, frontline managers usually have more power and experience a higher personal sense of power. This allows them to more easily influence their subordinates and achieve task goals [19,20].

Applying a manager-centric perspective, this study applies a job demands–resources (JD–R) model [21] to examine the potential effect of sense of power on emotional exhaustion. According to the JD–R model [21], first-line managers usually have high job demands (e.g., heavy workload, work stress, and emotional demands), but few job resources (e.g., rewards, decision authority, and social support). As a result, they will easily suffer physical and mental health problems such as emotional exhaustion [21,49,50]. As power-holders that powerless subordinates rely on in the organization, frontline managers are in charge of making professional and predictable decisions, and guide subordinates to increase organizational effectiveness [51]. The health impairment process of the JD–R model indicates that these onerous job demands will consume frontline managers’ vigorous resources, increase work strain, and result in emotional exhaustion [52]. The motivational process of the JD–R model notes that job resources (e.g., sense of power) provide support and assistance for individuals. This boosts work engagement and mitigates job demands and related physical and mental depletion [21,52]. In other words, sense of power is a crucial resource to elevate the wellbeing of frontline managers and to relieve their work stress and negative emotional state.

Specifically, frontline managers with an enhanced sense of power usually perceive that they control more valued resources and possess greater autonomy to carry out their occupational tasks. This provides them with more opportunities to address stressful situations and buffer the passive influence of job demands, alleviating their work pressure, and emotional exhaustion [53]. Moreover, frontline managers with a high sense of power will fully use their resources to cope with the requirements of their positions and concentrate on task-relevant information to achieve organizational goals. They are not easily distracted by outside interferences that reduce job resource consumption and are protected from suffering serious emotional exhaustion [54,55]. In contrast, frontline managers with a low sense of power are more easily affected by exterior constraints and social pressure [19]. Furthermore, high job demands cost considerable effort and energy, exposing them to strain, anxiety, emotional exhaustion, and other health problems [52]. In summary, a sense of power is a core resource for frontline managers to alleviate the influence of job demands on their work strain and to relieve emotional exhaustion. Hence, we hypothesize that:

**Hypothesis** **1.** Sense of power is negatively related to frontline managers’ emotional exhaustion.

### 2.3. The Mediating Role of Managerial Self-Efficacy

Self-efficacy refers to an individual’s belief in the ability to complete work in a specific situation [25,26]. Self-efficacy is a critical predictor of behavior; people devote significant effort to accomplish tasks once they perceive themselves able to achieve them. Previous researchers have studied self-efficacy in the fields of cognitive and social psychology, demonstrating strong relationships between self-efficacy and personal performance in organizations [56]. As a concrete form of self-efficacy, managerial self-efficacy is defined as the managers’ perception of their own capacity and the self-confidence that they are competent and can effectively conduct management tasks [27,28]. Prior work found that managerial self-efficacy is a significant predictor of supervisory performance (e.g., conduct more task-oriented leadership behavior), and is a critical need for leaders to fulfill their managerial responsibilities [27]. However, existing literature on self-efficacy has not examined the effect of sense of power on managerial self-efficacy. This study applies self-efficacy theory [25,26], proposing a cognitive path by which frontline managers with an elevated sense of power tend to have high managerial self-efficacy. This, in turn, enhances their emotional well-being.

Specifically, self-efficacy theory [25,26] posits that the initiation and persistence of behavior mainly depends on the judgment and expectations of behavioral skills and abilities, and the possibility of successfully addressing work requirements and challenges [57]. In other words, personal self-efficacy enhances when individuals perceive themselves as competent, effective, successful, and meritorious [27]. Accordingly, managers with heightened sense of power tend to believe that they have control over follower-valued resources and objects, and have confidence in their competence to implement their managerial roles. This enables them to effectively complete management work and increases their managerial self-efficacy [27,58]. In addition, previous studies found that supervisors who felt powerful held more confidence in making decisions [59], perceiving more personal control [23], solving management difficulties, and leading their followers [27]. This provides evidence that sense of power is positively correlated with managerial self-efficacy.

Past scholars have emphasized that self-efficacy is a primary determinant of stress (e.g., [60,61]). For example, Janjhua, Chaudhary, and Chauhan [62] found individuals who perceive stronger self-efficacy will experience less role stress caused by work demands. Moreover, self-efficacy theory [25,26] proposes that individuals with high self-efficacy tend to believe they can capably control their work, rarely worry about their failure to complete tasks, and rarely express negative attitudes toward their jobs [63]. This positive self-cognition provides individuals with continuous psychological resources (e.g., confidence) to energetically accomplish their work and enhances their emotional well-being. Consequently, frontline managers with elevated managerial self-efficacy are rarely affected by serious role stress and generally maintain confidence in their abilities to complete managerial tasks, avoiding the experience of emotional exhaustion. In conclusion, consistent with existing studies (e.g., [60]), we predict that managerial self-efficacy is inversely related to frontline managers’ emotional exhaustion.

Overall, from the perspective of social cognition and based on the theory of self-efficacy, a higher sense of power leads to more positive experiences, leading to higher managerial self-efficacy for front-line managers. This enables coping with different job demands, thus reducing emotional exhaustion. Thus, we hypothesize that:

**Hypothesis** **2.** Managerial self-efficacy mediates the negative relationship between sense of power and emotional exhaustion.

### 2.4. The Mediating Role of Affective Commitment

Becker [64] first proposed the concept of organizational commitment, which refers to the psychological state that employees remain in an organization as their investment and contribution to the organization accumulate. Meyer and Allen [65] developed a three-component model of commitment; the components include a desire (affective commitment), a need (continuance commitment), and an obligation (normative commitment). Affective commitment is defined as an individual’s emotional attachment to the organization so that the committed individual identifies with, is involved in, and enjoys organizational membership [32,66]. Prior meta-analyses have found that affective commitment is strongly correlated with outcomes such as stress and job satisfaction compared to other components (e.g., [67]). Mercurio [68] found that affective commitment is a central component of organizational commitment. This study, therefore, used affective commitment as a mediator through which frontline managers’ sense of power affects emotional exhaustion. Specifically, we applied social exchange theory [31] and proposed an affective path where sense of power strengthens frontline managers’ affective commitment, lessening their emotional exhaustion.

Social exchange theory [31] states that self-interested individuals form interdependent relationships through unspecified obligations. These obligations represent an economic exchange based on material resources and social exchange based on trust and reciprocity [31]. Social exchange is a bidirectional transaction that achieves mutual benefits, and includes two core characteristics: self-interest and interdependence [69,70]. Only social exchange can create feelings of personal obligation, gratitude, and trust [31]. Social exchange theory recognizes a core principle of reciprocity, where supervisors obtain a higher sense of power as the organization provides them with organizational status, authority, support, care, and other resources. This leads to an enhanced sense of obligation to return benefits to the organization. Frontline managers therefore reciprocate to the organization in the form of positive work results, benefitting the organization through personal efforts [71]. Satisfaction with individual needs and expectations (e.g., sense of power) lead frontline managers to actively develop affective commitment to the organization [72]. In addition, interdependence between frontline managers and organizations enhances frontline managers’ identification and trust in the organization, increasing affective commitment to the enterprise [70].

Frontline managers with high affective commitment have closer affective connections with the organization, have higher levels of recognition and participation, and are more willing to show positive work behaviors such as working hard to achieve organizational goals [32,73]. By further developing social relations with the organization, they tend to receive more support and resources, making them more competent on the job, alleviating stress and emotional exhaustion. Previous studies have supported this argument. For example, Schmidt [74] found that affective commitment is a valuable resource to promote personal well-being, and reduce personal anxiety and emotional exhaustion.

In summary, given the discussion above, social exchange theory holds that the higher the sense of power of frontline managers, the stronger the affective commitment will be to the organization. This connects them more closely to the organization and provides more resources to better meet job demands and reduce emotional exhaustion. Therefore, we hypothesize that:

**Hypothesis** **3.** Affective commitment mediates the negative relationship between sense of power and emotional exhaustion.

**Figure 1:**
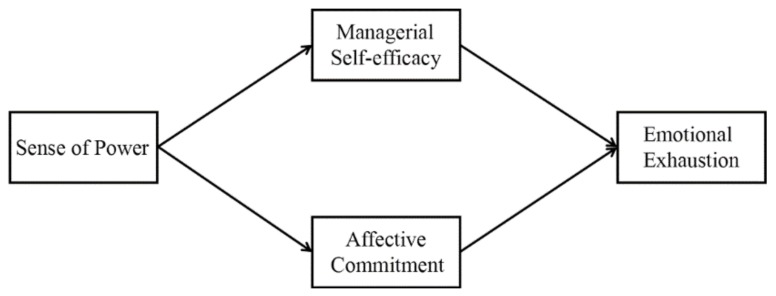
The proposed conceptual model.

Figure 1 presents the dual mediation model.

## 3. Sample and Methods

### 3.1. Participants and Procedures

Participants were on-the-job Master of Business Administration (MBA) students from a university in Southwestern China, working as frontline managers in different kinds of companies. We initially distributed paper questionnaires to 409 participants, informed them that the survey was aimed at the frontline manager and could continue to complete the survey if it was met, and received 227 complete matched and valid samples (response rate = 55.50%). Of the respondents, 52.86% of the sample was male, with an average age of 32.265 years (SD = 5.289). On average, they had worked at their companies for 6.080 years (SD = 4.948).

To control for common method biases [75], we conducted a three-wave survey, with a two-week interval between every two consecutive measurements. Specifically, participants were asked to report on their sense of power, demographic variables, and zhongyong at Time 1; affective commitment and managerial self-efficacy at Time 2; and emotional exhaustion at Time 3.

The survey was anonymous, but at the end of each survey, participants were asked to write down their phone numbers. The phone number was used as a label to match each participant’s three surveys. The telephone number was also used to reward participants with 10 yuan RMB in telephone fees, as a reward after each survey. To encourage continued participation, respondents were told that after the three surveys were completely matched, they would receive an additional 10 yuan RMB in telephone fees as a reward.

### 3.2. Measures

All study measures were translated into Chinese following translation and back-translation procedures [76].

Sense of power. We used an 8-item scale developed by Anderson et al. [17] to measure the sense of power. An example item is “I think I have a great deal of power.” A five-point Likert scale was adopted, from 1 (strongly disagree) to 5 (strongly agree). The Cronbach’s alpha was 0.920 for this survey.

Affective commitment. We assessed affective commitment using a 5-item scale developed by Gao-Urhahn, Biemann, and Jaros [77]. An example item is “I am glad to have joined this organization.” Each item was anchored by a five-point Likert scale, ranging from 1 (strongly disagree) to 5 (strongly agree). The Cronbach’s alpha was 0.940 for this survey.

Managerial self-efficacy. Participants were asked to assess their managerial self-efficacy using an 8-item scale developed by Fast et al. [28]. An example item is “I will be able to successfully overcome many challenges.” Responses were on a five-point Likert scale, from 1 (strongly disagree) to 5 (strongly agree). The Cronbach’s alpha was 0.931 for this survey.

Emotional exhaustion. Emotional exhaustion was assessed using a 3-item scale developed by Watkins et al. [78], which has been proven to be validated (e.g., [9,79]). An example item is “I feel exhausted when I think about having to face another day on the job.” A five-point Likert scale was used, from 1 (never) to 5 (very often). The Cronbach’s alpha was 0.871 for this survey.

Controls. Based on previous studies (e.g., [6,80,81]), we controlled for gender, age, and company tenure. Data collection was done in the context of Chinese culture. As such, to control possible interference with research conclusions, we also controlled for zhongyong, which many researchers regard as a core feature of Chinese culture (e.g., [82]). Previous studies have referred to zhongyong as the Confucian doctrine of the mean [83]. We assessed zhongyong using the six-item short version scale, adapted by Du, Ran, and Cao [84] based on Chiu’s [85] original scale. The assessment included a five-point scale from 1 (strongly disagree) to 5 (strongly agree). One example of an item is: “Everything has limitations, so it is not very good to exceed them.” The Cronbach’s alpha was 0.799 for this survey.

### 3.3. Ethical Statement

Based on institutional guidelines and national laws and regulations, no ethical approval was required for this research. This is because our study did not involve human clinical trials or animal experiments. We implemented steps to ensure that participants’ information was kept secure and private. In addition, all frontline managers participated on a voluntary basis. Verbal consent was obtained from each participant before the study began.

## 4. Results

### 4.1. Confirmatory Factor Analysis

To examine the distinctiveness of all the constructs (sense of power, zhongyong, managerial self-efficacy, affective commitment, and emotional exhaustion), we conducted a confirmatory factor analysis (CFA) using Amos 24.0. Table 1 shows that our hypothesized five-factor model (χ2 = 694.938, df = 395, χ2/df = 1.759, RMSEA = 0.058, CFI = 0.933, IFI = 0.933, TLI = 0.926) yielded a better fit than alternative models. This confirmed the distinctiveness of the five measures.

### 4.2. Descriptive Statistics and Correlation Analysis

We used the statistical software package SPSS 25.0 to analyze the study data. Table 2 contains the descriptive statistics with the means, standard deviations, and correlations of the studied variables. The table shows that sense of power is positively correlated with affective commitment (r = 0.444, *p* < 0.01) and managerial self-efficacy (r = 0.369, *p* < 0.01). Sense of power is negatively related to emotional exhaustion (r = −0.259, *p* < 0.01). Moreover, both affective commitment (r = −0.404, *p* < 0.01) and managerial self-efficacy (r = −0.360, *p* < 0.01) are negatively associated with emotional exhaustion.

### 4.3. Hypothesis Testing

We took three steps to test our hypotheses, following a procedure developed by Baron and Kenny [86]. First, we tested the impact of sense of power on emotional exhaustion. Model 3 in Table 3 shows that sense of power was negatively associated with emotional exhaustion (B = −0.319, SE = 0.084, *p* < 0.001), after controlling for the effects of gender, age, company tenure, and zhongyong. This result supported Hypothesis 1. Second, we examined the effect of sense of power on managerial self-efficacy and affective commitment. Models 1 and 2 in Table 3 shows that sense of power was positively correlated with managerial self-efficacy (B = 0.243, SE = 0.047, *p* < 0.001) and affective commitment (B = 0.502, SE = 0.077, *p* < 0.001), respectively. Third, we tested the impact of managerial self-efficacy and affective commitment on emotional exhaustion. Model 4 in Table 3 shows that managerial self-efficacy (B = −0.342, SE = 0.121, *p* < 0.05) and affective commitment (B = −0.290, SE = 0.073, *p* < 0.001) are significantly negatively related to emotional exhaustion, whereas sense of power had no significant impact on emotional exhaustion (B = −0.091, SE = 0.086, *p* > 0.05). Above all, the effect of sense of power on emotional exhaustion was mediated by managerial self-efficacy and affective commitment, thus supporting Hypothesis 2 and Hypothesis 3.

In addition, we applied the PROCESS macro in SPSS developed by Hayes and Preacher [87] to test the mediating effect. Concretely, a bootstrapping analysis (5000 samples) found that managerial self-efficacy had a significant mediating effect on the relationship between sense of power and emotional exhaustion (B = −0.083, 95% CI [−0.196, −0.016]). Similarly, affective commitment also significantly mediated the relationship between sense of power and emotional exhaustion (B = −0.145, 95% CI [−0.258, −0.060]). These results support Hypothesis 2 and Hypothesis 3.

**Figure 2:**
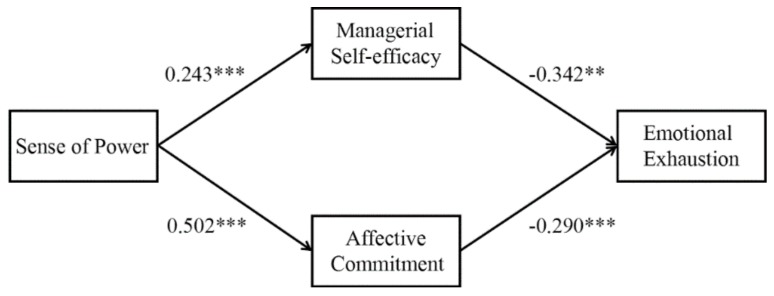
The results of regression in the full mediation model. Notes. * *p* < 0.05, ** *p* < 0.01, *** *p* < 0.001.

Figure 2 shows a summary of the regression results.

## 5. Discussion

Drawing on the JD–R model [21], we proposed and found support that frontline managers who perceive a greater sense of power tend to experience less emotional exhaustion. Furthermore, consistent with theories that propose that contextual factors affect behavior through the cognitive–affective states dual path (e.g., [88,89]), we found that managerial self-efficacy mediates the negative effect of sense of power on emotional exhaustion. Affective commitment also serves as a mediator.

### 5.1. Theoretical Implications

This research makes several critical contributions to the theory of and literature about emotional exhaustion and power. First, the study centered on frontline managers, testing the negative correlation between the sense of power and emotional exhaustion. This extends our understanding of antecedents of emotional exhaustion. Previous studies have largely drawn attention to the emotional exhaustion of employees or healthcare professionals (e.g., [90,91,92]). Fewer studies have concentrated on the importance of the frontline managers’ emotional exhaustion. As key personnel in organizations, frontline managers have more resources and power, and a greater sense of power compared to their subordinates [19,93]. However, there has been less research about how the sense of power affects emotional exhaustion. Our research, applying the JD–R model, demonstrates that frontline managers with an enhanced sense of power tend to believe that they have greater resources, autonomy, and authority to fulfill their management roles [27,53], and experience reduced emotional exhaustion.

Second, this study proposed a dual mediation model and underlying mechanism about how sense of power negatively and significantly affects emotional exhaustion through managerial self-efficacy and affective commitment, from the perspective of the cognitive–affective dual path. Specifically, self-efficacy theory [25,26] states that frontline managers with a greater sense of power tend to have high managerial self-efficacy. This is because they believe they are capable and competent to cope with management difficulties, lead their followers [27], and accomplish other managerial tasks [58]. This, in turn, decreases their fear of failing to finishing work, and the occurrence of emotional exhaustion. Additionally, within the framework of social exchange theory [31], frontline managers with a high perception of power usually have enhanced affective commitment, because they have established a stronger social exchange relationship with organizations. This boosts their satisfaction and identification with their enterprises and motivates them to complete work better to contribute to organizational effectiveness [32,73], alleviating emotional exhaustion. In short, revealing the cognitive and affective path contributes to an enriched understanding of the mechanism involved with emotional exhaustion.

Third, by revealing the mediating roles of managerial self-efficacy and affective commitment in the relationship between sense of power and emotional exhaustion, our research enriches an understanding of the consequences of sense of power, thus contributing to the power literature. Specifically, in contrast to previous studies focusing on the effect of power [94], power distance [92], and empowerment [1,14] on emotional exhaustion, this study concentrated on the frontline managers’ subjective perception of power and considered their sense of power to be a core resource for preventing emotional exhaustion. Moreover, managerial self-efficacy and affective commitment served as two bridges to link sense of power and emotional exhaustion. This helps indicate how sense of power plays its role, while also extending its outcomes to address managerial self-efficacy, affective commitment, and emotional exhaustion.

### 5.2. Practical Implications

Beyond the theoretical implications, the present study also highlights several important practical implications. First, organizations should focus on building a sense of power among frontline managers. The subjective perception of power is a critical resource for frontline managers to relieve their emotional exhaustion. This also affects collective outcomes (e.g., team performance) in organizations [58,95]. Therefore, it is essential to take measures that enhance the managers’ sense of power in the workplace. When initially selecting managers, it is important to judge whether candidates can draw on psychological resources such as psychological capital to increase their sense of power [19]. Importantly, organizations should provide more resources (e.g., autonomy and decision authority) for managers to effectively cope with job demands. This would further improve their perception of self-worth and sense of power.

Second, this study positioned managerial self-efficacy as a mediating variable linking the frontline managers’ sense of power and emotional exhaustion. There was a negative correlation between managerial self-efficacy and emotional exhaustion. Consistent with previous studies, a manager’s belief in the ability to perform managerial tasks well leads to lots of positive outcomes such as voice behavior [28] and engaging in task-oriented leadership behavior [27], and performing positive managerial job engagement [96]. Thus, organizations should commit to enhancing frontline managers’ managerial self-efficacy to improve their mental well-being. Specifically, Wood, Bandura, and Bailey [97] found that mastery experience is the most effective way to boost individuals’ self-efficacy. This should remind organizations to create positive conditions (e.g., enhancing sense of power and providing positive feedback [98]) that enable frontline managers to experience success, raising their managerial self-efficacy. The theory of self-efficacy [25,26] holds that, in addition to direct successful experience, positive indirect experience also helps improve self-efficacy. Organizations can create opportunities for frontline managers to exchange successful management experiences, and provide relevant sharing and training programs to improve the managerial self-efficacy of frontline managers.

Third, affective commitment appears to mediate the negative effect of sense of power on emotional exhaustion. This highlights the necessity of elevating the affective commitment of frontline managers to avoid the devastating outcomes of emotional exhaustion. In addition to fostering sense of power, organizations can apply other methods to enhance the affective commitment of frontline managers. Concretely, organizations can conduct high-commitment human resource practices (e.g., promoting role clarity and providing organizational support for managerial goals) to improve frontline managers’ organizational trust as well as their affective commitment to the organization [68,99]. Moreover, social exchange theory [31] indicates that organizations should work to meet the core needs of frontline managers to improve their emotional attachment to the organization, thereby improving their affective commitment.

### 5.3. Limitations and Future Research Directions

The current research makes significant theoretical and practical contributions to the fields of sense of power and emotional exhaustion. However, like all studies, there were some limitations that point to future directions for research. First, although we conducted a three-wave survey to alleviate concerns related to common method variance, this research used a cross-sectional design and adopted self-reported data. Future research would benefit from utilizing a longitudinal or experimental design to explore the causal relationship between the sense of power, managerial self-efficacy, affective commitment, and emotional exhaustion. Second, this study included a sample of frontline managers to test the theoretical model. To investigate the generality of our findings, future research should test the study results with samples of managers at all levels including middle and senior managers. Finally, this study collected data based on a Chinese cultural background and controlled the potential impact of the zhongyong. Our conceptual model, thus, may not hold true in other cultural contexts. Future studies are needed to determine whether the results can be applied to other cultures.

## 6. Conclusions

Drawing upon the JD–R model [21], this research proposed and examined a cognitive–affective dual mediation model of the relationship between sense of power, managerial self-efficacy, affective commitment, and emotional exhaustion, focusing on frontline managers. Our results confirmed that sense of power significantly alleviated frontline managers’ emotional exhaustion via managerial self-efficacy (cognitive path) and affective commitment (affective path). We hope that the current research will encourage future researchers to explore other interesting mechanisms mitigating emotional exhaustion from the perspective of the cognitive–affective dual path.

## Figures and Tables

**Table 1:** Confirmatory factor analyses in the study.

Models	χ^2^	df	χ^2^/df	RMSEA	CFI	IFI	TLI
5-factor model	694.938	395	1.759	0.058	0.933	0.933	0.926
4-factor model a	1010.987	399	2.534	0.082	0.863	0.864	0.850
4-factor model b	1468.046	399	3.679	0.109	0.760	0.762	0.739
3-factor model	1782.643	402	4.434	0.123	0.690	0.693	0.665
1-factor model	2814.028	405	6.948	0.162	0.460	0.463	0.420

Note. The 5-factor model is the basic hypothesized measurement model. In the 4-factor model a, sense of power and zhongyong were combined. In the 4-factor model b, affective commitment and managerial self-efficacy were combined. In the 3-factor model, sense of power and zhongyong were combined into one factor, and affective commitment and managerial self-efficacy were combined into the second factor. Finally, all the five variables were combined into one factor to form a 1-factor model. RMSEA = root mean square error of approximation; CFI = comparative fit index; IFI = incremental fit index; TLI = Tacker-Lewis index.

**Table 2:** Descriptive statistics, correlations, and reliability estimates.

Variables	M	SD	1	2	3	4	5	6	7	8
1. Gender	1.471	0.500	-							
2. Age	32.265	5.289	−0.266 **	-						
3. Company tenure	6.080	4.948	−0.087	0.622 **	-					
4. Zhongyong	4.088	0.466	0.068	0.053	0.125	(0.799)				
5. Sense of power	3.275	0.737	−0.143 *	0.211 **	0.078	0.190 **	(0.920)			
6. Affective commitment	3.580	0.915	−0.049	0.210 **	0.083	0.161 *	0.444 **	(0.940)		
7. Managerial self-efficacy	4.027	0.539	0.010	0.080	0.043	0.277 **	0.369 **	0.485 **	(0.931)	
8. Emotional exhaustion	2.464	0.909	0.016	−0.035	−0.039	−0.097	−0.259 **	−0.404 **	−0.360 **	(0.871)

Note. *n* = 227. M = mean; SD = standard deviation. Values on the diagonal represent Cronbach’s alpha (α). Gender: 1 = male, 2 = female. * *p* < 0.05, ** *p* < 0.01.

**Table 3:** Regression results for direct and indirect effects.

Variables	Dependent Variables			
	MSE	AC	Emotional exhaustion	
	Model 1	Model 2	Model 3	Model 4
**Controls**				
Gender	0.052(0.069)	0.081(0.114)	−0.017(0.124)	0.024(0.115)
Age	0.002(0.008)	0.029(0.014) *	0.008(0.015)	0.017(0.014)
Company tenure	−0.002(0.009)	−0.011(0.014)	−0.008(0.015)	−0.012(0.014)
Zhongyong	0.245(0.073) ***	0.156(0.120)	−0.086(0.131)	0.043(0.124)
**Independent Variable**				
Sense of power	0.243(0.047) ***	0.502(0.077) ***	−0.319(0.084) ***	−0.091(0.086)
**Mediators**				
MSE				−0.342(0.121) **
AC				−0.290(0.073) ***
R^2^	0.183	0.221	0.071	0.207
F	9.874 ***	12.547 ***	3.396 **	8.158 ***

Note. MSE = managerial self-efficacy; AC = affective commitment. The coefficients reported in the models are all non-standardized coefficients. * *p* < 0.05, ** *p* < 0.01, *** *p* < 0.001.
